# Unmet needs in the management of exacerbations of chronic obstructive pulmonary disease

**DOI:** 10.1007/s11739-020-02612-9

**Published:** 2021-02-22

**Authors:** Kiki Waeijen-Smit, Sarah Houben-Wilke, Antonio DiGiandomenico, Ulf Gehrmann, Frits M. E. Franssen

**Affiliations:** 1 Department of Research and Education, Ciro, Horn, NM 6085 The Netherlands; 2 NUTRIM School of Nutrition and Translational Research in Metabolism, Maastricht, The Netherlands; 3 Department of Respiratory Medicine, Maastricht University Medical Centre (MUMC+), Maastricht, The Netherlands; 4 Discovery Microbiome, Microbial Sciences, Biopharmaceuticals R&D, AstraZeneca, Gaithersburg, USA; 5 Translational Science and Experimental Medicine, Research and Early Development, Respiratory and Immunology (R&I), BioPharmaceuticals R&D, AstraZeneca, Gothenburg, Sweden

**Keywords:** Chronic obstructive pulmonary disease, Exacerbations, Biomarkers, Microbiome, Microbiota

## Abstract

Exacerbations of chronic obstructive pulmonary disease (COPD) are episodes of acute worsening of respiratory symptoms that require additional therapy. These events play a pivotal role in the natural course of the disease and are associated with a progressive decline in lung function, reduced health status, a low physical activity level, tremendous health care costs, and increased mortality. Although most exacerbations have an infectious origin, the underlying mechanisms are heterogeneous and specific predictors of their occurrence in individual patients are currently unknown. Accurate prediction and early diagnosis of exacerbations is essential to develop novel targets for prevention and personalized treatments to reduce the impact of these events. Several potential biomarkers have previously been studied, these however lack specificity, accuracy and do not add value to the available clinical predictors. At present, microbial composition and host-microbiome interactions in the lung are increasingly recognized for their role in affecting the susceptibility to exacerbations, and may steer towards a novel direction in the management of COPD exacerbations. This narrative review describes the current challenges and unmet needs in the management of acute exacerbations of COPD. Exacerbation triggers, biological clusters, current treatment strategies, and their limitations, previously studied biomarkers and prediction tools, the lung microbiome and its role in COPD exacerbations as well as future directions are discussed.

## Background

Chronic obstructive pulmonary disease (COPD) is a common, preventable, and treatable lung disease that is characterized by persistent airflow limitation and chronic respiratory symptoms [1]. Its main risk factor is cigarette smoking but environmental or occupational pollutions, prematurity, childhood infections, and genetic susceptibility may also contribute to the development of COPD [1]. Exposure to environmental risk factors is associated with chronic pulmonary inflammation and induces structural changes in the small airways and lung parenchyma [2–6]. These changes persist even after smoking cessation due to largely unknown mechanisms, although perturbation of the lung microbiota is suggested to play a role [7]. Moreover, the structural and inflammatory manifestations associated with COPD extend beyond the lungs [8]. Major symptoms of COPD are dyspnea, chronic cough, and sputum production [1] but extra-pulmonary manifestations such as fatigue [9] and exercise limitation [10] are also common. COPD is currently the third leading cause of death worldwide [1] and is associated with high disease and economic burden [1, 11]. In 2016, the disease claimed 3 million lives worldwide, of which 300.000 deaths occurred in Europe [12]. The direct healthcare costs of COPD are estimated to be €38.6 billion in Europe, excluding indirect costs such as COPD-related reduced workplace and home productivity [1]. Exacerbations of COPD, the main drivers of poor outcomes in the disease, are responsible for the greatest proportion of COPD-related healthcare costs [1]. Continued exposure to risk factors as well as the aging population are expected to contribute to an increased global COPD burden in the coming years [1].

### Exacerbations of COPD

While perceived respiratory symptoms are naturally fluctuating by day, week or season in patients with COPD [13], COPD exacerbations are part of the natural course of the disease and are defined as an acute worsening of respiratory symptoms that result in a need for additional therapy [1]. Exacerbations are complex events and are usually associated with increased airway inflammation, mucus production, increased sputum volume and purulence, cough and wheeze, marked gas trapping, and hyperinflation [4, 14]. These changes contribute to increased dyspnea, the key symptom of an exacerbation [1]. Prior exacerbations, cigarette smoking, low medication adherence, poor lung function and health status, and high blood eosinophil counts are well-recognized risk factors for COPD exacerbations [1, 15, 16]. Other risk factors include a history of heartburn or reflux [17], an increased ratio of the diameter of the pulmonary artery to the diameter of the aorta [18], a greater proportion of emphysema or airway wall thickness and chronic bronchitis [19], as well as low vitamin D (due to its immune-modulating role) [1]. Furthermore, reduced antibody production was recently shown to be associated with exacerbation risk [20]. Nevertheless, the susceptibility of an individual patient to exacerbate remains largely unknown, and timely prediction of these events in individual patients remains unsuccessful.

Exacerbations play a pivotal role in the progressive decline in lung function [21], reduced health status [22], low physical activity [23] and increased mortality risk [24] in COPD. Indeed, severe exacerbations, defined as those requiring hospitalization [1], have an in-hospital mortality rate of 10%, while another 26% dies within 1 year [25]. The risk of exacerbations is related to the severity of airflow limitation; approximately 40% of patients with moderate COPD experienced at least one exacerbation in the previous year while this percentage increases with an additional 10% respectively as the disease progresses to severe or very severe [17]. The identification of a specific ‘frequent exacerbator’ phenotype of COPD (i.e. ≥ 2 annual exacerbations) [1], 12% of all patients [17], resulted in a modification of disease classification in 2011 taking these events into account [19]. Moreover, one of the most recent advances in the understanding of COPD is the observation that distinct lung function trajectories may lead to its development [1, 26]. Patients of the normal maximally attained lung function trajectory seem to be at increased long-term risk of severe exacerbations [26]. Thus, it seems that it is not just airflow limitation, but also its preceding pathophysiology that predicts exacerbation risk.

### (Differential) exacerbation diagnosis

An increase in respiratory symptoms perceived by the patient forms the foundation of exacerbation diagnosis [1]. However, identification of exacerbations by patients as well as healthcare professionals is challenging. Indeed, approximately 75% of patients experience difficulties with the recognition of an exacerbation [27] and misdiagnosis by health care providers may occur due to the lack of clear diagnostic criteria [1]. As a result, under-recognition and under-reporting of these events, both by patients and clinicians, are common [28, 29]. Under-recognition and under-reporting are associated with slower recovery [27], which is associated with accelerated disease progression [30]. Worse health status and lung function are in turn related to exacerbation risk [31, 32], and once experiencing an exacerbation, the risk of subsequent exacerbations increases [17]. The result is a complex and vicious cycle.

Moreover, an increase in respiratory symptoms is not COPD specific and may mimic other acute events such as pulmonary embolism, pneumonia or heart failure which warrant alternative pharmacotherapies [16, 33]. It is estimated that 15–19% of all exacerbations are in fact exacerbation-like events, triggered by such differential acute conditions [16]. This highlights the importance of a thorough clinical assessment. Excluding alternative causes for increased symptoms has important clinical consequences, since patients with COPD often have multiple comorbidities [34, 35], which can both mimic and aggravate symptoms and outcomes of exacerbations [33, 36]. Indeed, COPD may be considered the pulmonary component of multimorbidity [37]. Because of such heterogeneity of the underlying disease, exacerbations should be diagnosed and treated taking their complexity into account. Therefore, over the last years, several proposals to come towards a precision medicine approach to COPD exacerbations have been published [33, 38]. Diagnosing COPD exacerbations should include assessment of vital parameters, laboratory testing, and electrocardiography. In addition, radiologic examinations enable the exclusion of differential cardiopulmonary conditions [1]. First, when differential diagnoses have been ruled out, the diagnosis of COPD exacerbation should be considered [1, 33]. Subsequently, the severity and trigger of the exacerbation should be assessed to determine the appropriate treatment setting and strategy [1].

### Exacerbation triggers and biological clusters

It is well recognized that exacerbations are heterogeneous events with respect to etiology and inflammation [14, 17, 39]. Most exacerbations have an infectious origin and are triggered by respiratory bacterial- and viral infections [40]. Of these infectious exacerbations, approximately half are triggered by bacterial infections whereas the other half is triggered by viral infections [40–42]. Furthermore, coinfection with both bacteria and viruses may also be observed [1, 41, 42]. These exacerbations are associated with more severe functional impairment and a longer duration of hospital stay [42, 43]. Also, seasonal exacerbation peaks have been reported and linked to interactions between bacterial and viral infections [43]. However, careful attention must be paid to the concepts of infection, colonization, coinfection, and co-colonization. As such, the co-existence of bacteria and viruses during exacerbations does not necessarily distinguish between acute infections or rather chronic co-colonization. In addition to infection driven exacerbations, eosinophilic inflammation may also trigger exacerbations [15, 39]. Eosinophilic inflammation can be observed in up to 20–40% of patients with COPD both during stable disease and exacerbations [41], and is associated with an increased risk of, especially eosinophilic [39], exacerbations [41]. Furthermore, environmental factors, e.g. air pollutants and meteorological effects such as outside temperature may also trigger exacerbations [43–45]. During the winter season there is an increased incidence of exacerbations, which may be driven by the increase of pathogens during this season [43]. The occurrence of exacerbations triggered by environmental factors is expected to rise in the coming years due to global climate change [45].

The heterogeneous etiology of exacerbations has played a pivotal role in the identification of distinct biological exacerbation clusters. To date, four different clusters have been proposed: the infectious ‘bacteria-predominant’ and ‘virus-predominant’ clusters as well as the ‘eosinophil-predominant’ and ‘pauci-inflammatory’ clusters [39]. The latter cluster is associated with limited inflammatory changes and has largely unknown pathophysiology [39]. Nevertheless, despite the fact that (acute) differential diagnoses should be ruled out [33] the contribution of comorbidities may be crucial in this latter cluster [46]. Therefore, increasing our understanding of the interactions and outcomes of co-occurring diseases in these events is key [37]. However, the exact etiology probably will remain unknown in a significant number of events [16]. Thus, there is a need for more sensitive microbiological, biochemical, radiologic and clinical diagnostics.

### Current exacerbation treatment strategies

Preventing exacerbations is one of the major aims in the management of COPD [1]. To reduce the health impact of these events in individual patients, early and accurate diagnosis followed by timely initiation of treatment is warranted. Indeed, adequate recognition of symptoms and early treatment is associated with faster recovery, reduced risk of hospitalization, and better health-related quality of life [43]. Since smoking is a major risk factor for exacerbations and hospitalizations, smoking cessation is essential in their prevention [47]. Other non-pharmacologic interventions associated with a reduced risk of exacerbations include enhancing physical activity [48], pulmonary rehabilitation [49] and self-management education [50]. Furthermore, vaccination (i.e. Influenza vaccine and *Pneumococcal* vaccines) is indicated in patients with COPD to reduce infection-related exacerbations [51].

Different maintenance pharmacotherapies exist to reduce symptoms, the frequency, and severity of exacerbations [1]. Depending on the patient’s treatable traits, treatments differing in their mechanism (and duration) of action, route of delivery, inhaler types, or combination treatments may be prescribed [1]. Common medications are bronchodilators (i.e. β2-agonists), anticholinergics (i.e. muscarinic antagonists), anti-inflammatory drugs (i.e. inhaled corticosteroids, oral glucocorticosteroids, and prophylactic antimicrobials), phosphodiesterase-4 inhibitors, and mucolytic agents [1, 14, 15]. At present, the Global Initiative for Chronic Obstructive Lung Disease (GOLD) strategy document recommends treating mild exacerbations with short-acting bronchodilators, and moderate and severe exacerbations with antibiotics and/or oral corticosteroids [1]. In addition to the previously mentioned pharmacotherapies, hospital management of exacerbations may include respiratory support such as oxygen therapy and (non-) invasive ventilation [1].

### Exacerbation cluster-specific treatment, are we there yet?

The majority of exacerbations are currently not treated according to their etiology but according to a one-size-fits-all approach. However, not all treatments may be necessary for all patients and for all exacerbations, and non-response to available treatments is common [41]. Hence, despite the available pharmacotherapies a significant proportion of patients with COPD continues to experience exacerbations, which is associated with a high burden of disease [1]. Only about 20–40% of all exacerbations have an eosinophilic inflammatory origin and respond well to systemic corticosteroids [41]. Therefore, several studies investigated whether an eosinophil-guided approach to treatment with systemic corticosteroids is safe and effective during exacerbations. Although this biomarker-directed approach was associated with reduced systemic corticosteroid exposure, non-inferiority regarding long-term outcomes of exacerbations is still debated [52]. Thus, the safety profile of this strategy warrants further research. While awaiting these results, an oral course of corticosteroids is recommended in patients with moderate or severe exacerbations [1].

With respect to the non-eosinophilic exacerbation clusters, those triggered by bacterial infections may require antibiotics [41]. Currently, increased sputum volume and/or purulence and dyspnea drive the decision for antibiotic treatment, although these assessments lack specificity [1, 33]. Several studies have investigated the role of biomarkers in guiding antibiotic therapy during COPD exacerbations. Using C-reactive protein (CRP), a non-specific marker of infections, or procalcitonin, a more specific marker of bacterial infections, to guide the decision of antibiotic treatment is not recommended for exacerbations of COPD [1]. The guidance of antibiotic treatment is important since adverse effects such as antibiotic resistance and increased microbial dysbiosis should be avoided [53], particularly in frequent exacerbating patients who often receive antibiotics [1]. Furthermore, exacerbations triggered by viral infections are often not treated etiologically, even though treatments do exist, e.g. antiviral vaccines or neuraminidase inhibitors to limit Influenza infections [41]. Finally, with respect to the pauci-inflammatory phenotype, no specific treatment strategies other than the available pharmacotherapies are currently available. In summary, exacerbation cluster-specific and personalized exacerbation treatment strategies are lacking and warrant further research.

### Biomarkers to predict COPD exacerbations

According to the National Institutes of Health, biomarkers are defined as ‘a characteristic that is objectively measured and evaluated as an indicator of normal biological processes, pathogenic processes or pharmacologic responses to a therapeutic intervention’ [54]. COPD exacerbation biomarkers may be used to improve early diagnosis, steer the characterization of disease severity, identify different phenotypes, serve as a prognostic tool predicting these events and perhaps most importantly steer targeted treatment strategies [41]. Biomarkers may also improve our understanding of the underlying pathophysiology of these acute events. Due to the heterogeneous nature of exacerbations, a biomarker must be specific and reflect the present exacerbation and not previous or subsequent events, particularly with regards to steering treatment decisions. Furthermore, biomarkers must be clinically implementable and therefore low-invasive, highly reproducible and cost-effective.

Several biomarkers potentially predicting exacerbations of COPD have previously been studied [33, 39, 41, 54–57]. Biomarkers of inflammation such as CRP, leukocytes, and fibrinogen are often elevated during exacerbations but are not specific for and predictive of exacerbations [55, 56]. Other blood markers such as surfactant protein D, correlate poorly with exacerbations [33]. Recently, a combination of elevated CRP, neutrophils and dyspnea revealed to discriminate best between the stable disease state and exacerbations [41]. In the Genetic Epidemiology of COPD (COPDGene) and SubPopulations and InteRmediate Outcome Measures in COPD (SPIROMICS) study, no blood marker added significantly to the prediction of exacerbations [57]. Rather, enlargement of the pulmonary artery diameter was associated with a three-fold increased exacerbation risk [18].

The composition of the pulmonary extracellular matrix is currently increasingly recognized to play a role in COPD exacerbations [2–5]. Increased levels of circulating extracellular matrix components such as collagen and hyaluronic acid have been shown during exacerbations [58, 59]. Indeed, inflammation is known to increase the turnover rate of the extracellular matrix and may explain the observed increased systemic levels of such markers during exacerbations [58]. Similarly, increased levels of endothelial-derived circulating extracellular vesicles were shown in patients with COPD, with further increases reported at exacerbations [60]. Nevertheless, it remains unknown whether systemic concentrations of these markers rise in the period leading up to an exacerbation. A potential predictive role remains to be confirmed.

Only few studies focused on the time window between exacerbation trigger and onset of deteriorating symptoms [61, 62]. The COPD and Seretide: a Multi-Center Intervention and Characterization (COSMIC) study used longitudinal diary card data to investigate the course of respiratory symptoms, daily peak expiratory flow (PEF) measurements, and daily rescue medication use to predict and analyze the natural course of exacerbations over 1 year [61]. Symptoms of dyspnea, cough and sputum, and nocturnal awakening steadily increased from 2 weeks prior to exacerbation, with a sharp rise during the last week. Importantly, the study showed that the course of symptoms was very similar between consecutive exacerbations in the same individual. Increases in symptoms and rescue medication use and decreases in PEF were associated with a higher risk to develop an exacerbation. However, predictive values were too low to warrant use in clinical practice [61]. Likewise, the patient-reported outcome Exacerbation of Chronic Pulmonary Disease Tool (EXACT) was effective at evaluating exacerbation severity but not at accurately detecting these events [62].

Taken together, no objectively measured COPD exacerbation biomarker is currently available. Previously studied biomarkers lack specificity, accuracy, and predictive value. Biomarkers to predict exacerbations form an important unmet need in the management of COPD and are warranted to improve early diagnosis and to steer treatment decisions. Due to the heterogeneous nature of exacerbations and the disease in general it is expected that different biomarkers might predict different exacerbation clusters [41, 63].

### Exacerbation cluster-specific biomarkers

Indeed, the identification of different biological exacerbation clusters has greatly contributed to the current concept of the heterogeneity of exacerbations and paved the way for more focused and in-depth studies. This includes the search for diagnostic biomarkers and the development of better-targeted treatments. As such, serum CXCL10 and sputum Il-1β have been linked to viral and bacterial-mediated exacerbations respectively, while the percentage of peripheral blood eosinophils was identified as the most sensitive and specific measure to determine sputum eosinophilia at exacerbation [39]. These results are potentially more accurate starting points for the individualized prediction and early identification of exacerbations through biomarkers. However, with regards to the bacteria-predominant cluster, not all bacterial strains induce Il-1β [33]. Therefore, future studies should assess potential alternative markers for such bacterial strains, and further increase our overall understanding of cluster-specific biomarkers. Overall, the stability and specificity of exacerbation (cluster) specific markers, their predictive and diagnostic value over time, their potential use for a subgroup of patients or rather personalized prediction, currently remain unclear.

### Exacerbation prediction tools

Multiple COPD exacerbation prediction tools have been developed over the years and are critically discussed in a systematic review of Guerra and colleagues [64]. Examples of measures included in these prediction models are the body mass index, airflow obstruction, dyspnea and severe exacerbations (BODEX) index, as well as other clinical predictors such as health-related quality of life and history of vascular disease [64]. Significant differences in designs and approaches can be observed, including the definition of exacerbations (i.e. event- or symptom-based approach), follow-up, and patient characteristics. Furthermore, many tools focus on hospital (re)admission instead of the prediction of an exacerbation per se. Overall, none of these models revealed to adequately predict exacerbations and are therefore not of clinical use [64]. Recently, the Acute COPD Exacerbation Prediction Tool (ACCEPT) was introduced to predict the individualized rate and severity of COPD exacerbations [65]. ACCEPT uses demographical and clinical variables, such as sex, smoking status, lung function parameters, and medication use, to estimate the 1-year exacerbation risk [65]. This tool has significant limitations as it does not differentiate between the different biological exacerbation clusters, neither does it include biomarkers or enable individualized prediction and early identification. A specific and accurate exacerbation prediction tool is therefore yet to be developed.

### The lung microbiome

There is a growing understanding of the lung microbiome in patients with COPD. The microbiome consists of symbionts; microbes with beneficial or neutral health effects, and pathobionts; potentially pathogenic microbes which under normal circumstances act as symbionts [66, 67]. These microbes are, of which pathobionts more efficiently, recognized through pattern recognition receptors such as toll-like receptors that are expressed on immune cells such as macrophages [68]. Respiratory symbionts affect host immunity via the production of metabolites such as short-chain fatty acids, sugars and amino acids [68], in addition, they may reduce pathobiont induced inflammation [66]. The microbiome is suggested to play a pivotal role in the pathogenesis of COPD as well as the onset of comorbidities and exacerbations [68, 69]. Conversely, factors such as the severity of airflow limitation, exacerbations, inflammation, and COPD-related lung medications, including antibiotics, corticosteroids and β2-agonists, may affect the lung microbial composition through local microenvironmental changes [66, 68].

The composition of the respiratory microbiota is variable and may change through migration, elimination, and growth of microbes, which is referred to as the transient microbiome [68]. Different types of microbes reside the microbiome including bacteria (i.e. bacteriome), viruses (i.e. virome), archaea (i.e. archaeome) and fungi (i.e. mycobiome) [68]. Important to note is that the microbiome refers to the complete set of genes, metabolites and microbes found in a particular environment, whereas the microbiota refers specifically to the microorganisms [68]. The local microenvironment depends on factors such as the type and presence of immune cells, pH, mucus, nutrients, and oxygen, and determines the composition of the local microbiome or local niches. Therefore, the site of sampling matters [68].

Indeed, the lung microbiota differs in function, colonization, and individual microbes from the gut microbiota [68]. Although, alterations and dysbiosis of the gut microbiota (e.g. cigarette smoking induced) have been linked to lung immunity [70] and respiratory diseases [71], suggestive of a gut-lung axis. As such, increased small intestine permeability was recently reported during severe exacerbations of COPD, in which hypoxia was suggested as an underlying mechanism [72]. The gut-lung axis is connected via the bloodstream and lymphatic system [68, 71] and acts bi-directionally [71]. Furthermore, direct micro-aspiration of the upper gastrointestinal microbiota may enter the lungs and affect the lung microbial composition [68]. The immune mechanisms as well as longitudinal changes and differences between the lung- and gut microbiota currently remain largely unknown.

### The respiratory microbiota in health and disease

**Fig. 1:**
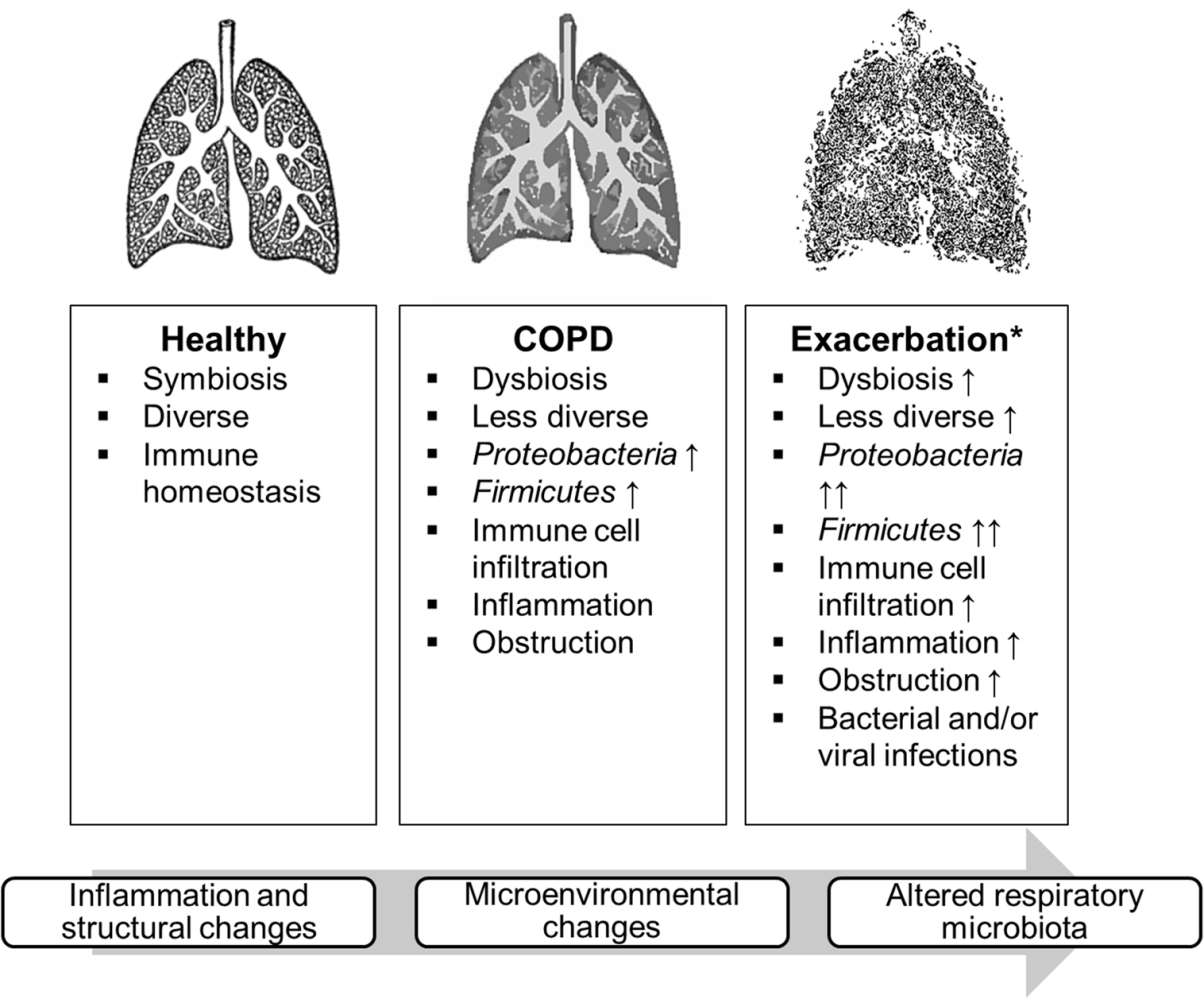
Common lung microbial characteristics of healthy individuals, patients with COPD at stable disease state and at acute exacerbations. *Not every exacerbation is characterized by these factors

Changes in microbial diversity have been associated with multiple chronic (respiratory) diseases [68, 71]. Indeed, the respiratory microbiota is suggested to be distinct between patients with COPD and healthy individuals [68, 73]. The core lung microbiota of healthy individuals is diverse and bacteria of the *Firmicutes, Bacteroidetes, Proteobacteria* and *Fusobacteria* phyla have been identified [68]. A healthy respiratory microbiota is characterized by microbial diversity, immune homeostasis, effective clearance of pathogens, and a state of symbiosis [53] whereas the lung microbiota of patients with COPD is less diverse and characterized by the abundance of specific phyla such as *Proteobacteria* [68] and *Firmicutes* [41, 43] (Fig. 1). Examples of common *Proteobacteria* are non-typeable *Haemophilus influenza* (NTHi), *Moraxella catarrhalis*, *Pseudomonas aeruginosa* and *Escherichia coli* [68]. Bacteria of the *Proteobacteria* phylum are associated with immune cell infiltration and therefore contribute to inflammation and obstruction of the airways [68] and are commonly identified at acute exacerbation [53]. Common exacerbation inducers of the *Firmicutes* phyla include *Streptococcus pneumoniae* [53, 68].

Less knowledge is available about the respiratory virome, although viral pathogens such as human rhinovirus (HRV) and influenza virus are also common in stable patients with COPD [43, 74]. Viral presence is known to increase the susceptibility to bacterial infections and is suggested to induce the development of COPD [67]. On the other hand, previous bacterial infections may determine immune responses to viral infections [75]. Moreover, dysbiosis of the virome may accompany the dysbiosis of the bacteriome [74]. With respect to cigarette smoking, no significant differences were recently observed between the respiratory microbiota of current and former smokers with COPD whereas significant differences were observed between patients with COPD and smoking controls [73]. To what extent cigarette smoking affects the composition of the respiratory microbiota, and whether smoking leads to the development of COPD through microbial changes, warrants further research.

### The lung microbiota and exacerbations of COPD

The altered lung microbiota of patients with COPD is suggested to mediate the susceptibility to exacerbations [40] and these alterations may further increase during exacerbations [68] (Fig. 1). Dysbiosis of the microbiota, particularly observed in individuals with eosinophilic inflammation, was shown to be associated with increased exacerbation severity and, amongst others, a greater symptom burden [76]. However, not every exacerbation is characterized by microbial dysbiosis [76] and microbial diversity was shown not to correlate with exacerbation frequency but rather with certain bacterial phyla [40]. As such, individuals with *Proteobacteria* predominant microbiota at stable disease state showed less microbial diversity and were shown to be more prone to suffer from future exacerbations and viral infections [40]. In addition, the exacerbation related length of hospitalization may be affected by the presence, or rather the absence of certain bacteria [77]. In this respect, the presence of *Proteobacteria* such as *Achromobacter* was related to increased length of hospital stay [77]. Finally, reduced microbial diversity has been linked to mortality [78] and antibiotic treatment failure [77] in patients with COPD.

Sputum analysis of patients with COPD at acute exacerbations revealed different bacterial communities between patients, as well as compared to stable states, highlighting the heterogeneity of COPD exacerbations [40]. In addition, some patients with COPD do not exacerbate although potential pathogenic bacteria are part of their microbiota. The mechanisms or factors responsible for this beneficial phenotype are currently still unknown [40]. Previously, an alternative concept of exacerbations caused by new microbial strains had been proposed. In this respect, exacerbations triggered by new bacterial strains, i.e. not residential bacterial strains, showed greater airway inflammation when compared to pathogen-negative exacerbations [41]. However, accumulating evidence now suggests that the balance between microbial populations rather than the infiltration of new strains determines host immune responses and that distinct microbial composition clusters are associated with distinct immune responses and different disease phenotypes [66, 68, 76]. For instance, the bacteria-predominant exacerbation phenotype shows an increased ratio of *Proteobacteria* to *Firmicutes* and is associated with a neutrophil predominant inflammatory response, whereas different microbial and inflammatory profiles are observed for the eosinophilic- and viral-predominant phenotypes [78, 79]. Furthermore, different clinical presentation patterns may be observed between the different inflammatory profiles of exacerbations [80].

With respect to the respiratory virome, viral infections may increase the risk of exacerbations although their role in exacerbation frequency is less significant [40]. Nevertheless, the presence of viruses worsens clinical outcomes during both the stable disease and exacerbations [81]. Possible explanations include the upregulation of bacterial adhesion molecules, damage to epithelial cells, and decreased mucociliary clearance, all of which are triggered by viral infections [68] and favor the persistence of pathogens. Commonly identified viruses at acute exacerbations are HRV, respiratory syncytial virus, influenza virus, and coronavirus [40, 53, 81]. Currently, it is still unknown whether viral infections promote the persistence of new infections or rather the outgrowth of pre-existing bacterial strains. While awaiting the results of a systemic review focused on the role of respiratory viruses in the stable disease state and during exacerbations in patients with COPD [81], no specific functional role of the virome has so far been identified other than inducing exacerbations [68].

Taken together, the lung microbiota regulates immune responses and the susceptibility to infections and exacerbations of COPD. Whether the changes in microbial composition are the cause or rather consequence of inflammation and exacerbations, is not clear yet. Moreover, despite the accumulating evidence of its importance, the clinical relevance is yet to be established. As techniques to investigate the lung microbiota become more available, future studies need to explore their application in clinical exacerbation management.

### Future directions

**Fig. 2:**
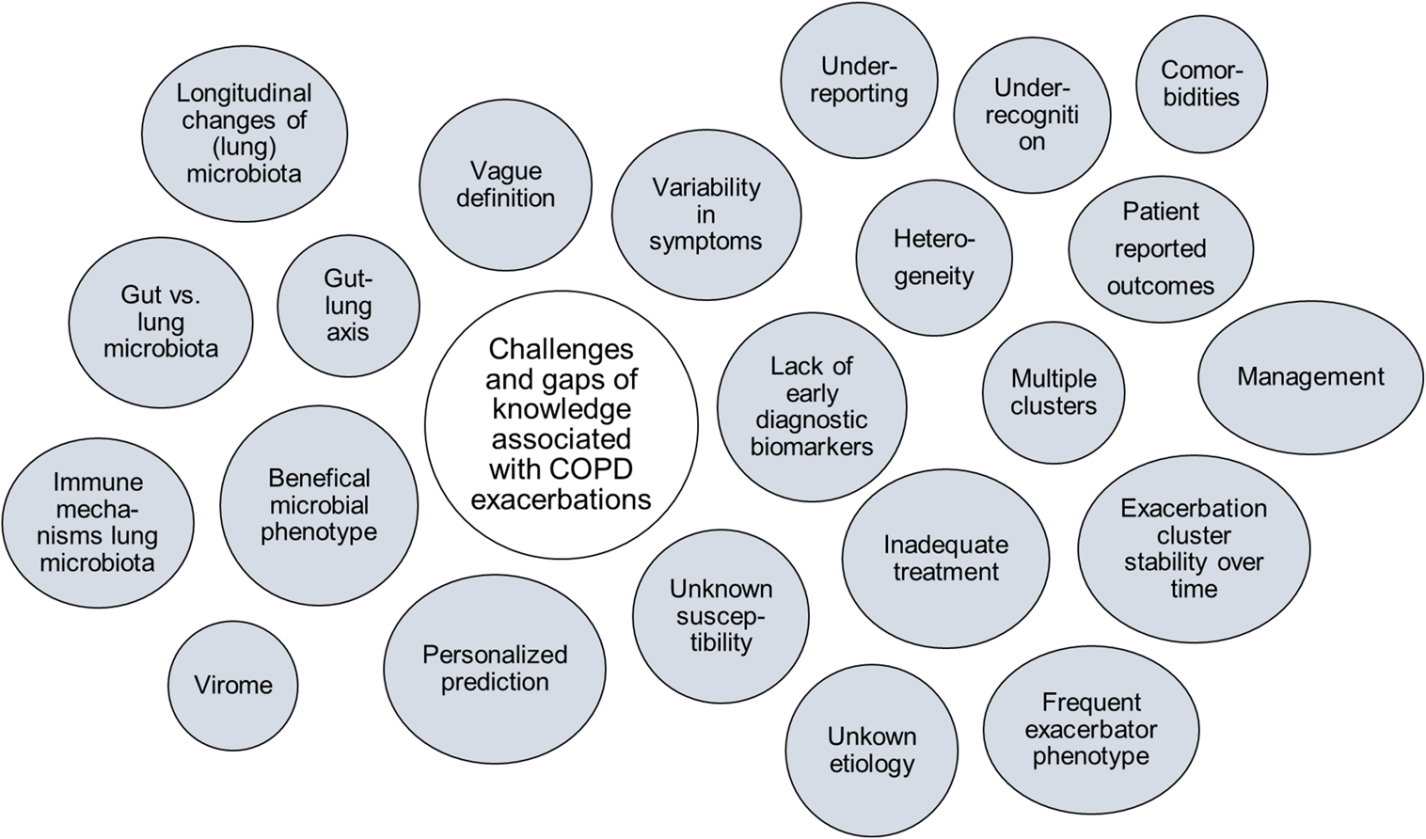
Current challenges and knowledge gaps associated with exacerbations of COPD

Many knowledge gaps, challenges, and unmet needs exist in the management of exacerbations of COPD (Fig. 2). Important challenges include, but are not limited to, the unknown etiology as well as susceptibility to exacerbations in a significant number of patients, the heterogeneity and biological clusters of exacerbations, the role of the respiratory microbiome and its constituents in the susceptibility to exacerbations, the unknown mechanisms responsible for a “beneficial” microbiotic phenotype (i.e. having a pathogenic microbiota although not exacerbating) as well as the immune mechanisms behind these phenotypes, and perhaps most importantly; the lack of predictive exacerbation biomarkers. A precision medicine approach is warranted in the prediction, prevention, diagnosis and treatment of these pivotal events to minimize their negative impact in patients with COPD. Biomarkers may guide this approach [33].

It is evident that multifactorial influences, e.g. demographics, pulmonary physiology, comorbidities, and genetic profile, play a role in the patient’s susceptibility to future exacerbations [15, 16] and these must be taken into account while searching for exacerbation biomarkers. Furthermore, future studies should focus on a broad(er) time window to assess whether biomarkers could already be detected when the exacerbation is triggered and well before the patient experiences a worsening of respiratory symptoms. Early biomarkers of exacerbations may be identified in blood [39] and nasal samples [82] whereas lung microbial composition and host-microbiome interactions can be assessed in sputum [39]. Omics and bioinformatics methodologies may be used to further increase our knowledge of the microbiome in patients with COPD [68].

Biomarkers based on the etiologic origin may steer more accurate treatment decisions. Although, it is important to note that the detection of the pathogenic origin does not necessarily distinguish between commensal entities or pathogens, nor does it distinguish a lower respiratory tract infection from an exacerbation [16, 41]. This highlights the importance of longitudinal follow-up and thorough clinical assessment for etiological origins to guide treatment decisions. Potential future directions for treatments may include active or passive vaccination against specific bacteria known to induce exacerbations, such as *Haemophilus* and *Moraxella*, to reduce their pathogenic and inflammatory burden, boost host defense responses, prevent secondary infections, and improve clinical outcomes in patients [68]. In addition, strategies to promote host immunity and growth of commensal microbes, such as pre- and probiotics [68] or pharmabiotics (i.e. live microbial therapeutics that use drug pathways) [83] might improve the management of exacerbations. Supplementation of probiotics in patients with cystic fibrosis for example reduced the frequency of pulmonary exacerbations as well as intestinal inflammation [68], but is unexplored in COPD. Taken together, more in-depth studies on exacerbation biomarkers are warranted before new treatment strategies can be developed.

## Conclusion

Exacerbations are complex and heterogeneous events with a central role in the burden and progressive course of COPD. There is a lack of identified biomarkers for accurate prediction and early diagnosis of these events in individual patients and of our understanding of longitudinal alterations in microbial composition and host-microbiome interactions in the stable state, at acute exacerbations, and during recovery. This is essential information for the development of novel antimicrobial and other therapeutic targets for (the different types of) exacerbations and subsequent personalized treatment. These challenges need to be addressed to reduce the future impact of these events, avoid ineffective treatment in individual patients, reduce healthcare utilization, and improve overall care for patients with COPD.

## Data Availability

Not applicable.
